# Uncommon Presentation of a Giant Splenic Epidermoid Cyst: A Report of a Rare Case

**DOI:** 10.7759/cureus.85419

**Published:** 2025-06-05

**Authors:** Moh'd Obeidat, Ruba Alsukour, Tareq Alzoubi, Saja Awaimreen, Tariq Bataineh, Hashem Abu Ain, Qais Abu-Mokleb, Elham Alsharaiah

**Affiliations:** 1 Department of General Surgery, Jordanian Royal Medical Services, Amman, JOR; 2 Department of Gynecology and Obstetrics, King Hussein Medical Center, Amman, JOR; 3 Department of Anesthesiology, King Hussein Medical Center, Amman, JOR; 4 Department of General Surgery, King Hussein Medical Center, Amman, JOR; 5 Department of Pathology, King Hussein Medical Hospital, Amman, JOR

**Keywords:** epidermal cyst, laparoscopy, spleen, splenectomy, vaccination

## Abstract

Primary splenic epidermoid cysts are extremely rare and typically discovered incidentally. The symptoms are variable, from an asymptomatic abdominal mass to severe abdominal pain. It is difficult to diagnose a primary splenic epidermoid cyst using imaging tests preoperatively, as it can appear similar to other cyst types. We report a 23-year-old woman who presented with left-sided abdominal pain. An advanced computed tomography (CT) scan revealed an 18 × 15 × 12 cm complicated splenic cyst, which was ultimately treated with a laparoscopic splenectomy, and the patient achieved a full recovery.

## Introduction

Splenic cysts are a unique medical condition. They are classified into primary and secondary splenic cysts according to the presence of the epithelial lining. Primary splenic epidermoid cysts are extremely rare, accounting for only 10% of all splenic cysts [1]. They are congenital and typically discovered incidentally. This condition predominantly affects children; however, it can also occur in adults, particularly young women [2]. The majority of splenic cyst cases are asymptomatic, but as the cyst grows in size, patients may experience various abdominal symptoms, including abdominal pain, an asymptomatic abdominal mass, nausea, and vomiting. Primary splenic epidermoid cysts are challenging to diagnose and distinguish from other types of cysts preoperatively using imaging modalities. Splenectomy is still the most accepted treatment of choice in cysts larger than 5 cm, but some prefer to consider conservative management [3,4]. Here, we report a case of splenic epidermoid cyst that was successfully treated with laparoscopic splenectomy.

## Case presentation

A 23-year-old woman with no previous medical illness presented to our general surgery clinic with a one-year history of intermittent left-sided abdominal pain, which worsened over the past two months. She denied nausea, vomiting, fever, changes in bowel habits, rectal bleeding, appetite loss, or trauma. Her vital signs were normal. Clinical examination revealed splenomegaly and mild upper abdominal tenderness on deep palpation. Her laboratory test results were within normal limits (Table 1).

**Table 1:** Laboratory tests

Test name	Patient's serum level	Normal range	Unit
White blood cells	5.7	3.4-9.6	10^3^/µL
Red blood cells	5	3.92-5.13	10^6^/µL
Hemoglobin	12	11.6-15	g/dL
Hematocrit	35.6	35.5-44.9	%
Platelets	237	157-371	10^3^/µL
Chloride	103.2	95-108	mmol/L
Alanine aminotransferase (ALT)	13.9	≤41	U/L
Aspartate aminotransferase (AST)	14.5	≤37	U/L
Creatinine	0.59	0.5-1.2	mg/dL
Blood urea nitrogen (BUN)	10.4	6-20	mg/dL
Glucose serum	88	70-110	mg/dL
Sodium	139	135-153	mEq/L
Potassium	4.36	3.5-5.5	mEq/L
Calcium	9.58	8.4-10.5	mg/dL
Phosphorus	3.65	2.5-4.5	mg/dL
Uric acid	3	2.7-6.1	mg/dL
Albumin	48	35-55	g/L
Amylase	80	28-100	U/L
Total bilirubin	0.305	0.1-1.2	mg/dL
Direct bilirubin	0.094	0.01-0.3	mg/dL
*Echinococcus* antibodies	Negative	Negative: <1/160 titer; equivocal: 1/160 titer; positive: >1/320 titer	

**Figure 1:**
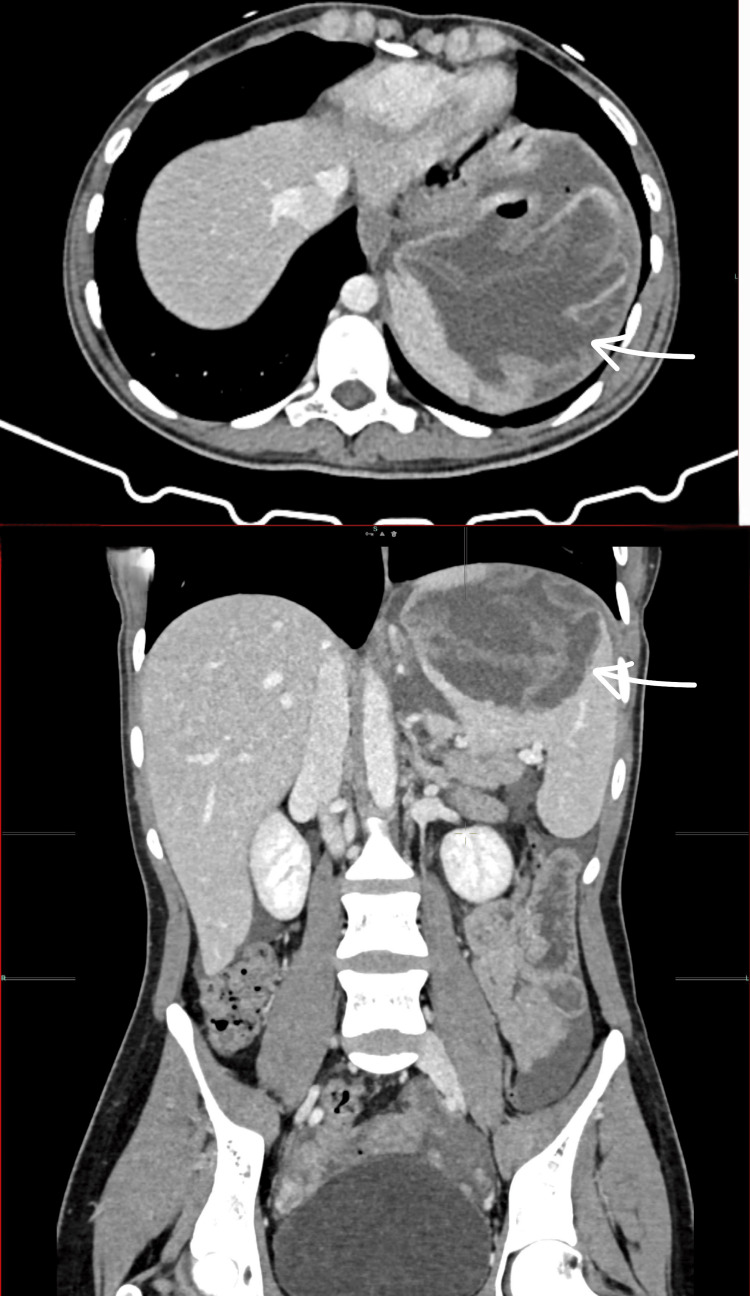
Two CT scout films of the patient at presentation showing a large complicated splenic cyst (18 × 15 × 12 cm) CT: computed tomography

A computed tomography (CT) scan revealed a large fluid density lesion (18 × 15 × 12 cm) in the upper pole of the spleen containing multiple gas locules extending into the left subphrenic region, with thin floating membranes. Moderate free fluid was present in the mid-upper abdomen, paracolic gutters, and pelvis (Figure 1).

**Figure 2:**
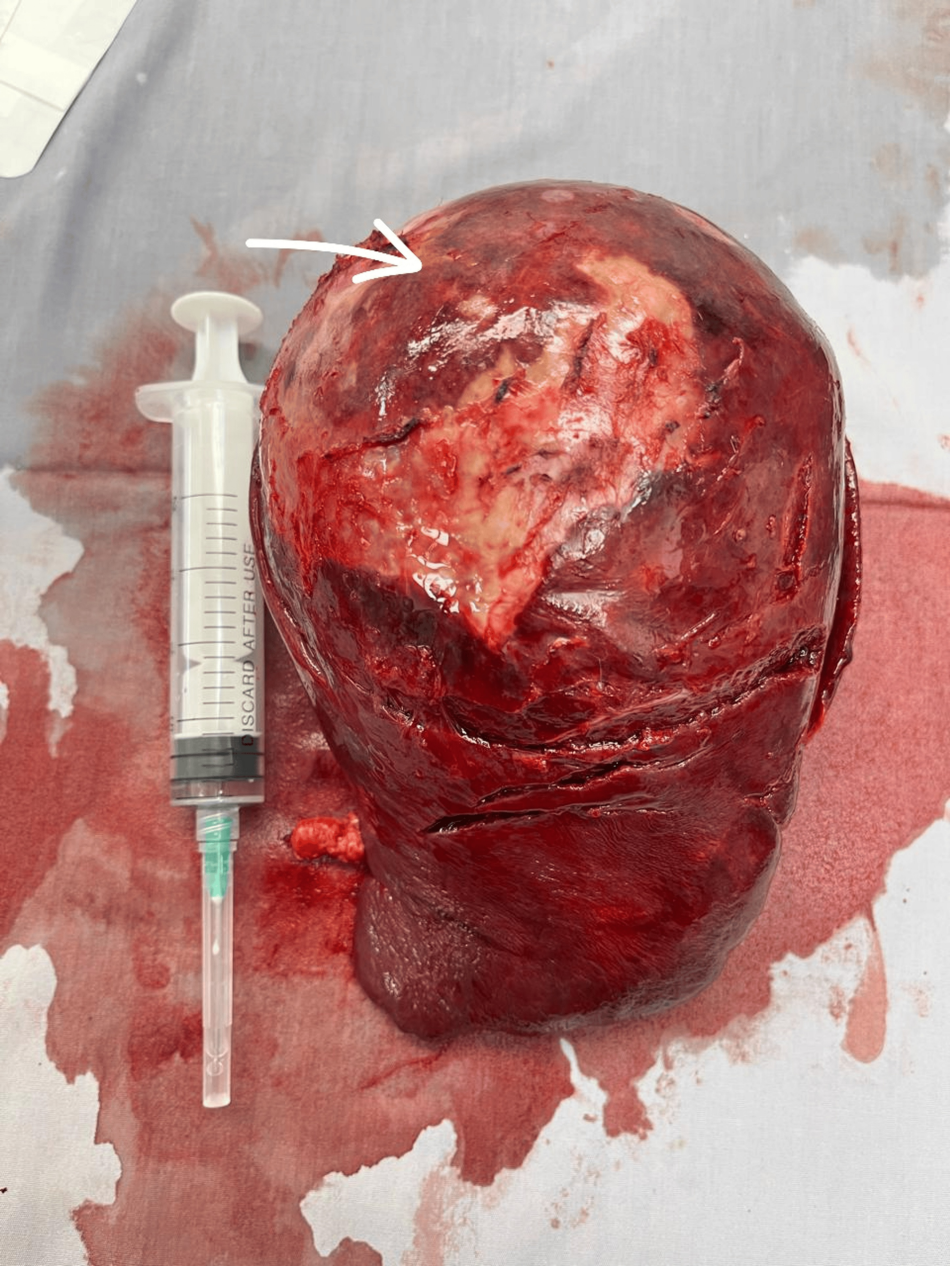
Intraoperative view of the spleen and the cyst

Based on these findings, an initial diagnosis of a complicated hydatid cyst of the spleen was made. Treatment options were discussed, and the patient initially chose a less invasive puncture, aspiration, injection, and re-aspiration (PAIR) procedure, which was performed successfully. She was discharged three days later with stable vital signs and normal laboratory tests. However, during her follow-up two weeks later, she reported persistent left-sided abdominal pain and insisted on surgical intervention. One week later, after obtaining informed consent and explaining potential complications, the patient underwent a laparoscopic splenectomy. The spleen was extracted through a Pfannenstiel incision (Figure 2).

**Figure 3:**
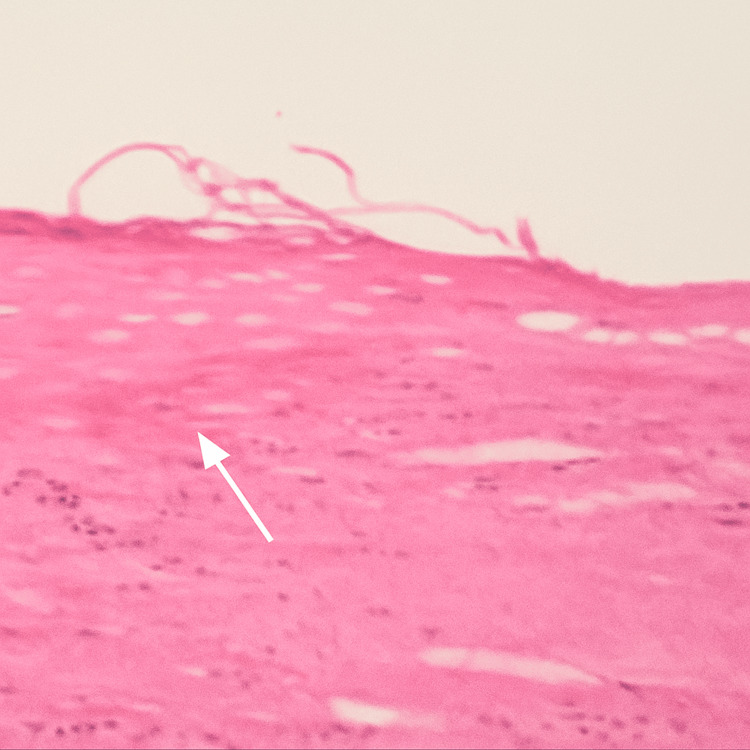
Splenic epithelial (epidermoid) cyst At low-power magnification, the cyst is lined by keratinized stratified squamous epithelium with an underlying fibrous wall and no skin adnexa

She recovered well and was discharged two days postoperatively. At her two-week follow-up, she was asymptomatic with a healed wound. She received vaccinations against pneumococcal, meningococcal, and *Haemophilus influenzae* infections. Pathological examination revealed a spleen measuring 10.5 × 15.5 × 17 cm with an intact cyst (10 × 13 × 14 cm) containing hemorrhagic fluid and compressing the splenic parenchyma. The inner cyst surface was smooth and whitish with dusky areas. Histopathological examination revealed that the cyst wall was fibrous, showing acute and chronic inflammation with focal hemorrhagic infarction. The cyst was lined by squamous epithelium, consistent with a benign splenic epidermoid cyst (Figure 3). The patient achieved complete recovery with no recurrence during follow-up.

## Discussion

The spleen is a fist-sized organ located in the upper left side of the abdomen, lateral to the stomach and posterior to the left ribs. It is held in place by four ligaments. The spleen plays a crucial role in the management of red blood cells and the immune system. Splenic cysts are extremely rare, with an estimated incidence of 0.07% [4]. According to Martin [5], these cysts are divided into two main categories: type I (primary or true) cysts, which have an epithelial lining and can be of parasitic origin (usually *Echinococcus granulosus*) or non-parasitic origin [1,4], and type II (secondary or false) cysts, which lack an epithelial lining. Non-parasitic type I cysts are further subdivided into congenital and neoplastic types. Congenital cysts include epidermoid, dermoid, and endodermoid cysts, while neoplastic cysts of the spleen include hemangiomas (the most common type) and lymphangiomas. Splenic epidermoid cysts are exceptionally rare, accounting for only 10% of all splenic cysts [1,4,6]. They are believed to originate from the entrapment of peritoneal mesothelial cells within the splenic parenchyma during embryogenesis in intrauterine life, although the precise mechanism remains unclear [1,4,6-8]. Splenic epidermoid cysts affect children; however, they can also manifest in adults, particularly in young women [9,10].

The clinical presentation of the splenic epidermoid cyst is variable. It may present as an asymptomatic left-sided abdominal mass found incidentally during regular follow-up or as an acute abdomen due to the rupture of the cyst [2,4]. Other non-specific symptoms may include nausea, vomiting, constipation, dyspnea, and urinary symptoms [2,7,10].

Diagnosing splenic epidermoid cysts can be challenging because there are no specific laboratory tests or imaging studies tailored for this condition. Ultrasonographic features of epidermoid cysts and hydatid cysts are similar, typically presenting as well-defined, solitary cystic lesions with or without septations and wall calcification [2,4,8]. Advanced computed tomography (CT) scans can provide a clearer picture regarding intra-cystic fluid, internal septations, or calcifications. CT scans can accurately identify the location, size, and adjacent structures of the cyst, making CT scanning the gold standard for preoperative planning [4,8]. On magnetic resonance imaging (MRI), splenic cysts show high signals on T1- and T2-weighted images. T1 signal intensity varies with cyst content, increasing with cholesterol, proteins, or blood products [7,10]. The histopathological features are distinctive and essential for the definitive diagnosis of primary splenic epidermoid cysts. These cysts are lined by keratinizing stratified squamous epithelium, similar to that of the skin. Their walls are typically thick and fibrotic and may exhibit signs of chronic inflammation and occasional calcification. In contrast to dermoid cysts, epidermoid cysts do not contain adnexal structures such as hair follicles, sebaceous glands, or sweat glands [5,11].

The treatment of splenic cysts depends on various factors, including the size of the cyst, the location of the cyst, the presence of symptoms, the patient's age, associated comorbidities, and the patient's choice. All splenic cysts larger than 5 cm or symptomatic should be managed surgically, either through open surgery or laparoscopically [2,3,7,8,10]. The aim of surgical intervention is to prevent complications and recurrence [10]. Different types of surgery have been mentioned in the literature, including splenectomy, partial splenectomy, cystectomy, marsupialization, and cyst decompression [1,4]. Due to overwhelming sepsis, the preservation of the spleen is highly recommended, especially for young patients, and for that reason, some surgeons consider more conservative management such as percutaneous drainage, aspiration, and the injection of sclerotherapy [1,2,8].

## Conclusions

Splenic epidermoid cyst is a rare surgical condition. Preoperative diagnosis is challenging due to non-specific symptoms and the absence of distinctive features on imaging modalities. A CT scan can be helpful in localizing the cyst and identifying adjacent structures. Splenectomy remains the treatment of choice for splenic cysts larger than 5 cm or symptomatic cysts. However, less invasive techniques (including partial splenectomy or cyst excision) may be considered as a treatment option for young patients with small or noncomplicated cysts to preserve splenic function. All splenectomized patients should receive vaccinations against pneumococcal infections, meningococcal infections, and *Haemophilus influenzae* to reduce the risk of overwhelming postsplenectomy infection.

## References

[1] Gray TT, Patel V, Timpone M, Felux K (2022). Primary splenic epidermoid cyst: a case report. Cureus.

[2] Alshammari S, Alshenaifi S, Alfawaz F, Alkanhal A, Alsaif F (2021). A 16-year-old Saudi boy with a symptomatic large splenic epidermoid cyst. Am J Case Rep.

[3] Guilbaud T, Portier F, Camerlo A (2019). Conservative laparoscopic approach in a large splenic epidermoid cyst. Updates Surg.

[4] Samarakoon LB, Si Min Goh S, Cheong YL, Ong LY (2018). Massive splenic epidermoid cyst in a child treated with laparoscopic partial splenectomy - case report and review of literature. Proc Singap Healthc.

[5] Martin JW (1958). Congenital splenic cysts. Am J Surg.

[6] Ramia JM, de la Plaza Llamas R, López-Marcano AJ, Valenzuela Torres JD, García Gil JM (2017). Laparoscopic partial splenectomy for a splenic epidermoid cyst. Cir Esp.

[7] Vuyyuru S, Kharbutli B (2017). Epidermoid cyst of the spleen, a case report. Int J Surg Case Rep.

[8] Rana AP, Kaur M, Singh P, Malhotra S, Kuka AS (2014). Splenic epidermoid cyst - a rare entity. J Clin Diagn Res.

[9] Chen YY, Shyr YM, Wang SE (2014). Erratum to: epidermoid cyst of the spleen. J Gastrointest Surg.

[10] Vo QD, Monnard E, Hoogewoud HM (2013). Epidermoid cyst of the spleen. BMJ Case Rep.

[11] Macheras A, Misiakos EP, Liakakos T, Mpistarakis D, Fotiadis C, Karatzas G (2005). Non-parasitic splenic cysts: a report of three cases. World J Gastroenterol.

